# Loss of Centromere Cohesion in Aneuploid Human Oocytes Correlates with Decreased Kinetochore Localization of the Sac Proteins Bub1 and Bubr1

**DOI:** 10.1038/srep44001

**Published:** 2017-03-13

**Authors:** Julie Lagirand-Cantaloube, Cendrine Ciabrini, Sophie Charrasse, Alice Ferrieres, Anna Castro, Tal Anahory, Thierry Lorca

**Affiliations:** 1 Université de Montpellier, Centre de Recherche de Biologie Cellulaire de Montpellier, CNRS UMR 5237, 1919 Route de Mende, 34293 Montpellier Cedex 5, France; 2 Unité de Cytogénétique DPI, CHU Arnaud de Villeneuve, 34295 Montpellier Cedex 5, France; 3 Unité d’AMP DPI, CHU Arnaud de Villeneuve, 34295 Montpellier Cedex 5, France; 4 Département de Gynécologie Obstétrique, Equipe Médecine de la Reproduction, CHU Arnaud de Villeneuve, 34295 Montpellier Cedex 5, France

## Abstract

In human eggs, aneuploidy increases with age and can result in infertility and genetic diseases. Studies in mouse oocytes suggest that reduced centromere cohesion and spindle assembly checkpoint (SAC) activity could be at the origin of chromosome missegregation. Little is known about these two features in humans. Here, we show that in human eggs, inter-kinetochore distances of bivalent chromosomes strongly increase with age. This results in the formation of univalent chromosomes during metaphase I (MI) and of single chromatids in metaphase II (MII). We also investigated SAC activity by checking the localization of BUB1 and BUBR1. We found that they localize at the kinetochore with a similar temporal timing than in mitotic cells and in a MPS1-dependent manner, suggesting that the SAC signalling pathway is active in human oocytes. Moreover, our data also suggest that this checkpoint is inactivated when centromere cohesion is lost in MI and consequently cannot inhibit premature sister chromatid separation. Finally, we show that the kinetochore localization of BUB1 and BUBR1 decreases with the age of the oocyte donors. This could contribute to oocyte aneuploidy.

Aneuploidy is the leading cause of congenital birth defects^1^ and is the main cause of poor pregnancy outcome in *in vitro* fertilization (IVF) protocols. However, although aneuploidy importantly contributes to birth defects and pregnancy loss^2^, little is known about the underlying molecular mechanisms. Results from early studies demonstrate that aneuploidy is mostly caused by errors during maternal gamete meiosis and that these errors increase with maternal age^1^,^3^.

Chromosomal missegregation in oocytes can be induced by different mechanisms. The first mechanism is failure to recombine and correctly locate crossovers and, consequently, to maintain chromosome connections^1^. More than 10% of oocytes contain at least one bivalent without DNA crossover^4^. The second mechanism involves the premature loss of sister chromatid and sister inter-kinetochore (ITK) cohesions. Chromosome cohesion is maintained by cohesin complexes that contain two subunits of the structural maintenance of chromosome (SMC) family (SMC1 and SMC3 in somatic cells, and RAC and STAG3 in germ cells) and the kleisin subunit SCC1/RAD21 (REC8 in germ cells)^5^,^6^,^7^,^8^. These complexes form a ring structure that surrounds sister chromatids and centromeres. At the metaphase I (MI)-anaphase I transition, degradation of cyclin B and securin allows the activation of separase, a protease that will then promote the cleavage of phosphorylated REC8 and induce the separation of sister chromatids. Centromere cohesion is maintained until metaphase II by the action of shugoshin (SGO) that, through binding to PP2A-B56, promotes dephosphorylation of REC8 that becomes resistant to cleavage by separase^9^. At the metaphase II-anaphase II transition, bipolar tension on sister kinetochores induces PP2A-B56 removal, REC8 phosphorylation and then cleavage by active separase.

In oocytes, aneuploidy is mostly the result of segregation errors during MI and their frequency increases with age^10^,^11^,^12^, possibly due to age-related decrease of sister chromatid cohesion during the dictyate stage of prophase I^13^,^14^. A defect in cohesion of chromosome arms that are distal to crossover sites could result in a shift of chiasmata placement and premature bivalent separation, leading to the presence of univalent chromosomes during MI. However, contradictory data about the presence^15^ or absence^16^ of univalents during MI in aged mouse oocytes have been reported. Besides chromosome arm cohesion, loss of sister centromere cohesion also could be involved in age-related egg aneuploidy. Sister kinetochores must be unified during MI to ensure their correct co-segregation. In mouse oocytes, bipolar attachments require a MI-specific sister kinetochore structure and are only achieved after several rounds of error correction, suggesting that homologous chromosome bi-orientation is error-prone^17^,^18^. Loss of sister centromere cohesion with age could disrupt the kinetochore structure, impair monopolar binding and facilitate stable, but incorrect bipolar attachment of sister kinetochores^15^,^19^. Indeed, several studies using aged mouse^18^,^20^,^21^ and human oocytes^21^,^22^,^23^ reported increased sister IKT distances during meiosis I that result in merotelic attachments (i.e., a single kinetochore is bound to microtubules from both spindle poles) and aneuploidy^15^. If the spindle assembly checkpoint (SAC) does not detect these incorrect attachments, anaphase I onset will not be prevented^24^,^25^,^26^. The SAC is a safeguard mechanism to avoid premature chromosome segregation before correct kinetochore binding to the spindle. In human mitotic cells^27^,^28^, SAC activity delays anaphase onset until all chromosomes are correctly attached to the spindle. Unattached kinetochores promote the assembly of an inhibitory complex that is called mitotic checkpoint complex (MCC) and is formed by the SAC proteins MAD2, BUBR1 and BUB3^29^,^30^. Once assembled, the MCC binds to CDC20, the anaphase-promoting complex (APC) activator, to inhibit the ubiquitination and degradation of cyclin B and securin, and ultimately to delay anaphase onset. The MCC is generated in a super-complex, called the KMN network formed by the proteins KNL1, MIS12 and NDC80, at the kinetochore interphase contacting with the microtubules^31^,^32^. SAC activation in unattached kinetochores is dependent on the binding of the SAC kinase MPS1 to the KMN subunit NDC80 at early mitosis. MPS1 is then activated by autophosphorylation and promotes KNL1 phosphorylation^33^,^34^,^35^,^36^. This results in the formation of a docking site for BUB3 that in turn brings about BUBR1, BUB1 and MAD1/MAD2, resulting in MCC formation^37^,^38^,^39^,^40^. Interestingly, MPS1 binds to the NDC80 domain that also contacts microtubules, suggesting that, when microtubules are correctly attached to NDC80, they prevent MPS1 binding to the kinetochore and inactivate the SAC^27^.

SAC role in regulating mitosis timing has been largely described in human cells. Without the SAC, mitosis is accelerated and chromosome missegregation occurs^41^. Results obtained in mouse oocytes demonstrate that loss of SAC components leads to strong acceleration of meiosis I, severe chromosome missegregations and sterility^42^,^43^,^44^. However, the SAC is not very efficient in meiosis, compared with mitosis, and the presence of one or few incorrectly attached kinetochores with reduced IKT tension escapes checkpoint detection in mouse oocytes^24^,^45^,^46^. A general decline of SAC protein levels in aged oocytes in mice and humans has been observed^47^,^48^,^49^,^50^. Similarly, in mouse oocytes a reduction of the kinetochore localization of the SAC protein MAD2 with age has been reported^16^. However, although SAC activity reduction could play a prominent role in maternal age-associated aneuploidy induced by loss of chromosome arms or IKT cohesion, neither the kinetochore localization of SAC proteins, nor SAC activity has ever been investigated in human oocytes.

Here, we assessed IKT cohesion and the SAC in human oocytes. Specifically, we investigated whether the IKT distance of bivalents and of sister chromatids in a cohort of human oocytes changes with maternal age. We next analysed the localization of the SAC proteins BUB1 and BUBR1 during meiosis and whether this localization was dependent on MPS1 activity. Finally, we investigated whether BUB1 and BUBR1 kinetochore localization was affected by IKT cohesion and maternal age.

## Results

### Meiotic stage distribution of oocytes from young and older women

Oocytes (n = 226) (Supplementary Fig. 1) were obtained following ovarian stimulation treatment for IVF and were staged at retrieval and recovery (3 h after retrieval) by contrast microscopy. They were classified as GV, MI or metaphase II (MII) according to the presence of the germinal vesicle (GV), the absence of both GV and polar body, and the presence of the polar body, respectively. Depending on their stage, they were: (1) frozen for western blot analysis (n = 40 MII oocytes), (2) immediately fixed or shortly cultured (n = 93) or (3) cultured for 24 hours without any treatment to follow meiotic progression (n = 73), or (4) cultured for 24 hours with reversine or DMSO (n = 20). Oocytes were then fixed and used for immunofluorescence and confocal microscopy analysis (Supplementary Fig. 1).

**Figure 1:**
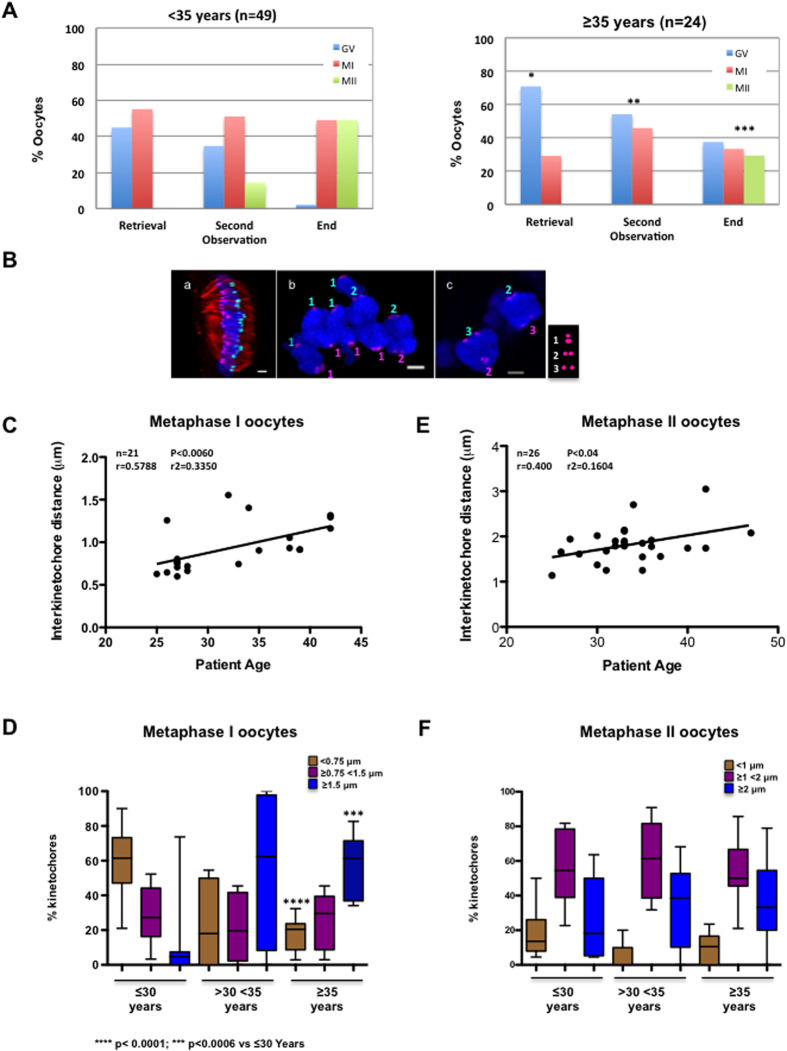
Inter-kinetochore distance in bivalents increases with maternal age. (**A**) Oocytes were analysed by confocal microscopy after retrieval, after 3 hours (Second Observation) and at the end of culture for 24 h (End) and classified as germinal vesicle (GV), metaphase I (MI) and metaphase II (MII) oocytes. The percentage of oocytes at the different meiotic stages according to the maternal age (<35 or ≥35 years) is shown. The oocyte meiotic stage distribution was significantly different in women <35 versus women ≥35 years of age; *p < 0.032; **p < 0.045 and ***p < 0.0046 (Kruskal-Wallis test). (**B**) Deconvolved images of a MI-arrested oocyte immunostained with anti-tubulin (red) and anti-CREST (magenta) antibodies (a) were analysed with Imaris for automated spot detection. Spots corresponding to kinetochores from homologous chromosomes are depicted in light blue and pink. (b and c) Deconvolved images from zoomed zones of different MI-arrested oocytes stained with anti-tubulin (red) and anti-CREST antibodies (Magenta). Sister kinetochore distances were measured using Imaris. The light blue and pink numbers indicate the kinetochore distance (1: inter-kinetochore distance <0.75 μm, 2: between 0.75 μm and 1.5 μm and 3: >1.5 μm). Scale bar: 2 μm. (**C**) Distribution of the inter-kinetochore distance of 21 MI oocytes relative to the donors’ age (25 to 42 years). (**D**) Box and whisker diagram showing the kinetochore distribution (median, minimum and maximum values) based on their inter-kinetochore distances (<0.75 μm, ≥0.75 and <1.5 μm and ≥1.5 μm) and according to the patients’ age (≤30 years, ≥30 and <35 years, and ≥35 years). Statistical analysis performed using the unpaired two-tailed Student’s *t* test. (**E**). Distribution of the inter-kinetochore distance of 26 MII oocytes relative to the donors’ age (25 to 42 years). (**F**) Like for (**D**) except that the inter-kinetochore distances in MII oocytes were classified as <1 μm, ≥1 and <2 μm and ≥2 μm.

Meiotic resumption was investigated in the oocytes with a 24 h-incubation (n = 73) at retrieval, recovery (3 h after retrieval; second observation) and after 24 h-incubation (end) by contrast microscopy (Fig. 1A). At retrieval time, 70.8% oocytes from older (≥35 years) women (n = 24) were classified as GV and 29.2% as MI, whereas 55.1% oocytes from younger (<35 years) women (n = 49) were in MI and only 44.9% in GV (significantly lower than in older women, p < 0.032). Oocytes from younger women rapidly matured from GV-MI to MI-MII during recovering time (3 hours after retrieval; second observation) and 49% of oocytes reached MII after 24 h incubation (End). Conversely, in older women, only 29.2% of oocytes were in MII and 37.5% were still in GV after 24 h incubation, suggesting that maturation is delayed in oocytes from older women. Results were not affected by the cause of infertility or stimulation protocol used.

### Inter-kinetochore distance in bivalents during metaphase I increases with maternal age

To investigate the mechanisms responsible of aneuploidy in human oocytes, first IKT distances were measured in MI and MII oocytes after immunofluorescence analysis with anti-tubulin (red) and anti-CREST (magenta) antibodies and acquisition of high-resolution z-stacks by confocal microscopy (Fig. 1B). IKT distance measurement in deconvolved images with Imaris (see Materials and Methods) allowed dividing sister kinetochores in three groups, based on the IKT distance <0.75 μm (Fig. 1B, 1 in panel b), between 0.75 μm and 1.5 μm (Fig. 1B, 2 in panel b and c) and >1.5 μm (Fig. 1B, 3 in panel c). Analysis of the mean IKT distance in MI oocytes according to the women’s age (Fig. 1C) showed that this distance increased with age. Specifically, the mean ± SD sister kinetochore distance was 0.73 ± 0.32 μm in women up to the age of 30 and 1.2 ± 0.34 μm in women older than 30 years (p < 0.006, Student’s *t* test). Moreover, analysis of the distribution of sister kinetochores according to the three IKT distance groups and the patients’ age (≤30 years, between 30 and 35 years and ≥35 years) showed that in MI oocytes from ≤30-year-old women, 60.15% of sister kinetochores had an IKT distance lower than 0.75 μm, 28.92% between 0.75 μm and 1.5 μm and only 10.92% higher than 1.5 μm (Fig. 1D). In 30 to 35-year-old women, 22.67% of sister kinetochores had an IKT distance below 0.75 μm, 21.18% between 0.75 μm and 1.5 μm and 56.16% above 1.5 μm. Finally, in women older than 35 years, IKT distance dramatically increased with most of sister kinetochores showing distances between 0.75 μm and 1.5 μm (27.33%) or higher than 1.5 μm (55.41%), suggesting a decrease of centromere cohesion in oocytes from aged women.

In MII oocytes, analysis of the mean IKT distance relative to the donor’s age indicated that split kinetochores were increased also in MII (Fig. 1E). Conversely, the IKT distance distribution in MII oocytes was not significantly different in the three age groups (Fig. 1F). These results must be interpreted cautiously because oocytes were not subjected to cold treatment to visualize k-fibres and, in the used conditions, the identification of sister chromatid pairs was difficult in some cases.

### The presence of univalents in MI oocytes and of single chromatids in MII oocytes increases with maternal age

**Figure 2:**
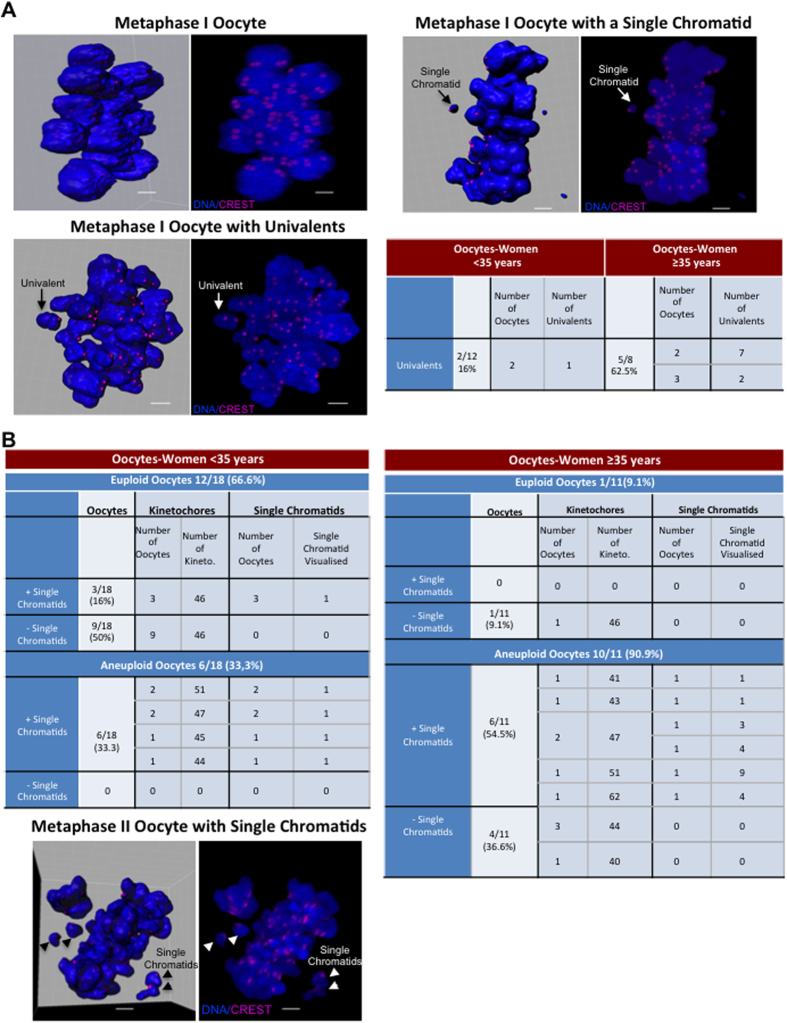
The presence of univalents in metaphase I (MI) oocytes and of single chromatids in metaphase II (MII) oocytes increases with maternal age. (**A**) Three MI oocytes with normal bivalent distribution (upper left panels) or with univalents (lower left panels) or single chromatids (upper right panels). Spots were automatically detected (left panels) with Imaris in deconvolved images of oocytes immunostained with Hoechst (blue) and anti-CREST (magenta) antibodies (right panels). The table shows the number of oocytes displaying univalents in oocytes from young (<35 years) and older (≥35 years) women. (**B**) Tables showing the number of kinetochores and single chromatids present in MII oocytes from younger (<35 years) and older women (≥35 years). Bottom, MII oocyte with single chromatids (representative image). Scale bars, 5 μm.

Loss of chromatid arms and centromere cohesion result in the disintegration of bivalents into univalents during MI^15^,^22^ and in the separation of sister chromatids at MII^16^,^20^ and this contributes to aneuploidy. Therefore, the presence of univalents and single chromatids in MI and MII oocytes was checked after immunofluorescence staining. In MI oocytes, the number of oocytes with univalents as well as the number of univalents per oocyte increased with maternal age (Fig. 2A and Videos 1 and 2). Specifically univalents were observed only in 16% (2 out of 12) of MI oocytes from women under 35 years and in 62.5% (5 out of 8) of MI oocytes from women over 35 years. Moreover, two oocytes from older women had a single chromatid instead of a univalent, as revealed by the smaller chromatin mass and the presence of one unique kinetochore (Fig. 2A and Video 3). This finding is in agreement with the results by Hodges and colleagues who showed the presence of single chromatids in MI oocytes following loss of centromere cohesion in SMC1β-deficient female mice^51^.

Then, to investigate whether the presence of isolated sister chromatids in MI oocytes could be linked to aneuploidy, kinetochores were quantified in MII oocytes (Fig. 2B). This analysis showed that overall, 55.1% MII oocytes (16 of 29) were aneuploid (Tables of Fig. 2B). Single chromatids could be detected only in three euploid oocytes compared with 12 aneuploid oocytes. When analysed according to the women’s age, 90.9% of MII oocytes from older patients were aneuploid compared with 33.3% in the younger group (Fig. 2B, right and left tables respectively). In oocytes from young women, one isolated sister chromatid was detected in 16% of euploid oocytes compared with 33.3% in aneuploid oocytes. Conversely, 54.5% of aneuploid oocytes from older women had from one to nine single chromatids (Video 4). Moreover, 83.3% of aneuploid MII oocytes with single chromatids in both age groups contained an odd number of kinetochores. This suggests that in these oocytes, at least one single chromatid results from abnormal kinetochore bi-orientation and sister chromatid separation at MI. All these findings suggest that decreased chromatid and centromere cohesion during MI in aged oocytes could result in disintegration of bivalents into univalents and in the premature separation of sister chromatids. This could contribute to age-related aneuploidy.

### Distribution of the SAC proteins BUB1 and BUBR1 during meiosis in human oocytes

Decreased centromere cohesion results in merotelic attachments in human oocytes^21^,^22^,^23^. In mitotic somatic cells, these improper attachments are corrected by the SAC at anaphase onset. Correct localization of BUB1 and BUBR1, two main SAC components, at kinetochores is essential for SAC signalling^33^,^36^. As little is known about BUB1 and BUBR1 expression/localization and about SAC functionality in human oocytes, immunofluorescence analysis of oocytes was performed using anti-tubulin, -CREST, -BUB1 and -BUBR1 antibodies. The specificity of the anti-BUB1 and -BUBR1 antibodies was checked by immunofluorescence analysis (Supplementary Fig. 2) and by immunoprecipitation followed by western blotting (Supplementary Fig. 3A) in human U2OS and HeLa cells, respectively. The secondary antibody non-specific background also was checked by immunofluorescence analysis in human oocytes (Supplementary Fig. 3B).

**Figure 3:**
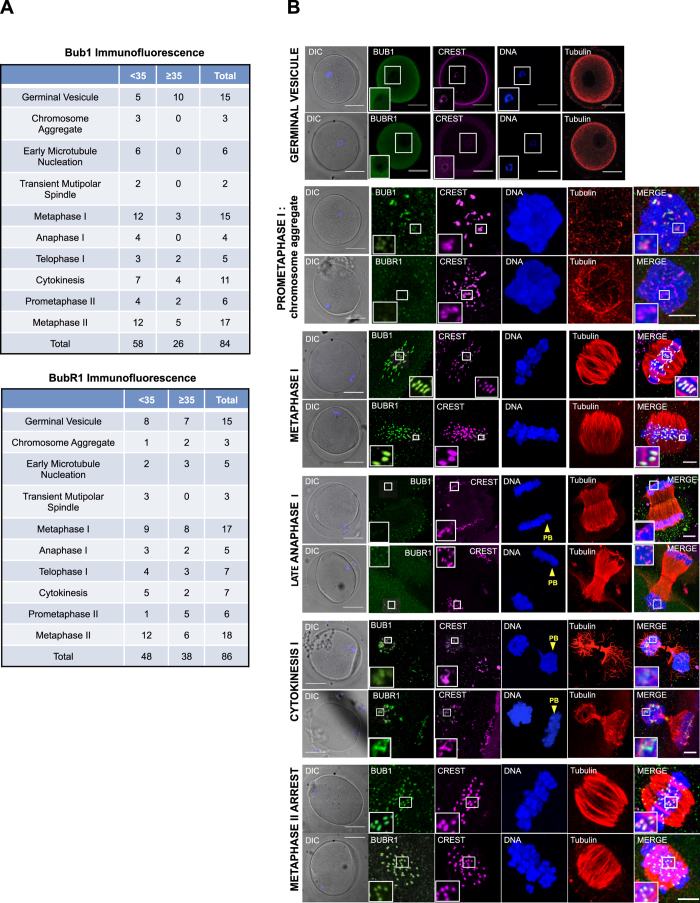
Distribution of the SAC proteins BUB1 and BUBR1 during meiosis. (**A**) The tables show the numbers of oocytes at different meiotic stages from <35 and ≥35-year-old women used for immunostaining with anti-BUB1 -BUBR1, -CREST and –tubulin antibodies. DNA was stained with Hoechst. (**B**) Representative confocal images of oocytes immunostained with anti-BUB1, -BUBR1, - CREST and -tubulin antibodies (one z-plan) during the different phases of meiosis. Scale bars, 50 μm, and 5 μm for zoomed images. DIC, differential interference contrast microscopy; PB, polar body.

The localization of BUB1 and BUBR1 was then analysed by confocal microscopy in oocytes classified in ten different stages on the basis of morphological meiotic changes, as previously described^22^ (Supplementary Fig. 4A) and according to the women’s age (Fig. 3A). BUB1 and BUBR1 localization at each meiotic phase were comparable in the two age groups. Both BUB1 and BUBR1 displayed a diffuse cytoplasmic localization throughout meiosis. However, the localization of a fraction of these two proteins changed during the various meiotic stages. Specifically, BUB1 and BUBR1 were diffuse in the cytoplasm of GV oocytes and did not co-localize with CREST. This indicates that during prophase these two proteins do not localize at kinetochores (Fig. 3B). BUB1 and BUBR1 co-localized with CREST from prometaphase I to MI. At prometaphase I, when chromosome aggregate was detected, the signal was weak for BUB1 and undetectable for BUBR1. However, their kinetochore localization was clearly detected when microtubules started nucleation (Supplementary Fig. 4B) and when they formed a transient multipolar spindle (Supplementary Fig. 4C). During this meiotic phase, spindle are very unstable and completely unstructured in early prometaphase and progress towards a multipolar spindle at the end of prometaphase. At MI, when spindle fibres align the chromosomes on the metaphase plate, BUB1 and BUBR1 signal shape changed from round to elongated probably due to kinetochore tension promoted by its binding to spindle microtubules (Fig. 3B). Meiotic spindles were visualized at this stage as flat barrel-shaped structures with unfocused spindle poles and they acquired their focused bipolar appearance at anaphase onset (Supplementary Fig. 5A).

In agreement with previous results obtained in mitotic human cells^52^, BUBR1, but not BUB1, signal at kinetochores decreased and rapidly disappeared at anaphase onset (Supplementary Fig. 5A and Fig. 3B). Conversely, BUB1 signal at kinetochores disappeared only during late anaphase (Fig. 3B). At telophase I, BUB1 was localized to the midbody microtubule and BUBR1 to the midbody ring (Supplementary Fig. 5B, white arrowheads). Moreover, BUB1 and BUBR1 were also detected, albeit weakly, at some kinetochores and to a lesser extent in the polar body (Supplementary Fig. 5B, zoom). At cytokinesis, both BUB1 and BUBR1 localized again at kinetochores (Fig. 3B).

During prometaphase II, chromosomes were normally distributed on the bipolar meiotic spindle, whereas undercondensed chromosomes were disposed on a disorganized meiotic spindle in the polar body (Supplementary Fig. 5C, yellow arrows). BUB1 and BUBR1 were recruited again at the kinetochores in late prometaphase II (Supplementary Fig. 5C) and MII (Fig. 3B) and, to a lesser extent, at kinetochores of the polar body (Supplementary Fig. 5C). In some oocytes, BUBR1 was detected also at the spindle poles during prometaphase II (Supplementary Fig. 5C, white arrowheads). Finally, BUB1 and BUBR1 were maintained at kinetochores of MII-arrested oocytes bound to a barrel-shaped MII meiotic spindle (Fig. 3B).

### Kinetochore localization of BUB1 and BUBR1 in human oocytes is dependent on MPS1 activity

**Figure 4:**
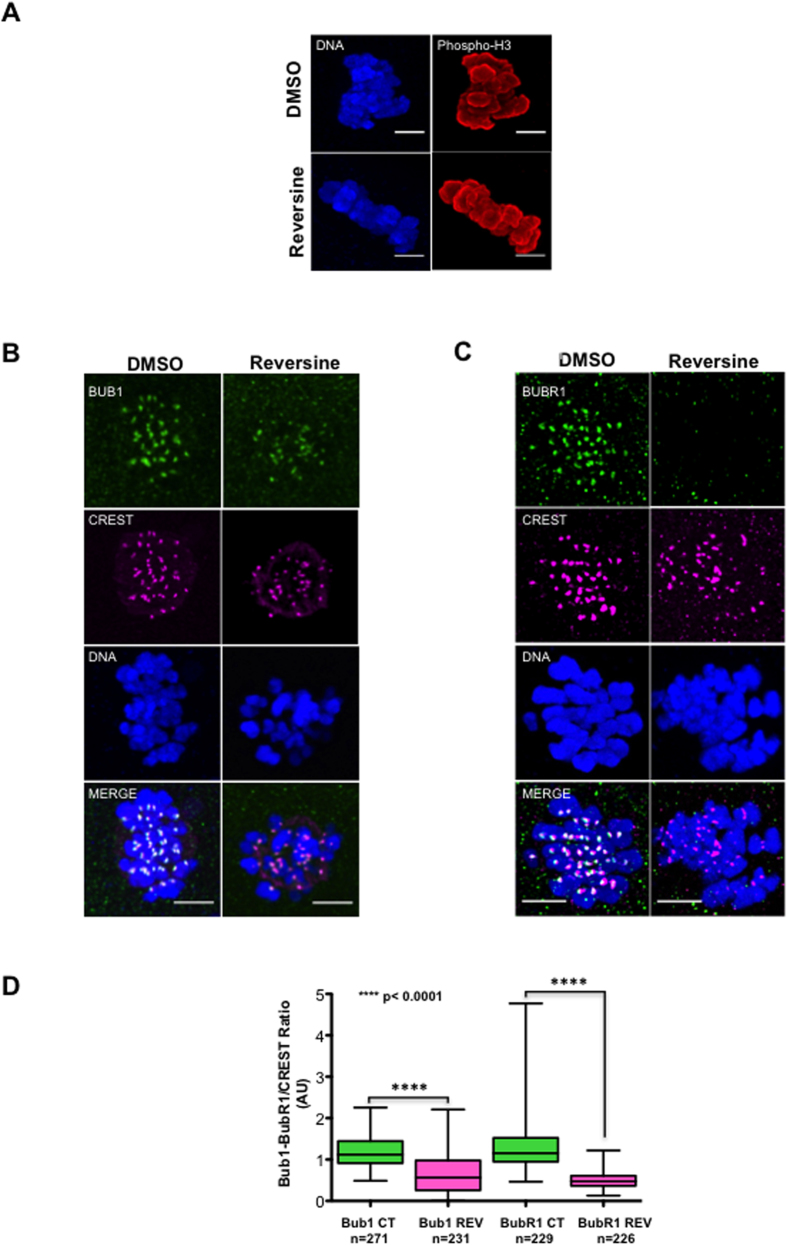
In human oocytes, BUB1 and BUBR1 kinetochore localization requires MPS1 activity. (**A**) Metaphase II oocytes were incubated (reversine) or not (DMSO) with the MPS1 inhibitor reversine for 30 min, stained with the anti-phosphorylated H3 on Ser10 antibody (Phospho-H3) and Hoechst and imaged by confocal microscopy. (**B**,**C**) Representative images of metaphase II oocytes incubated or not with reversine and then immunostained using anti-BUB1, -BUBR1 and -CREST antibodies and analysed by confocal microscopy. DNA was stained with Hoechst. Scale bars, 5 μm. (**D**) Box and whisker diagram showing the median, minimum and maximum values of the BUB1/BUBR1 to CREST immunofluorescence signal ratios of each kinetochore from oocytes incubated (REV) or not (CT) with the MPS1 inhibitor reversine for 30 min. Significance was assessed with the unpaired two-tailed Student’s *t* test.

BUB1 and BUBR1 localization at kinetochores is required for SAC functionality. Previous reports indicated that this localization is dependent on the activity of the MPS1 kinase^33^,^36^,^53^. To determine whether the signalling pathway that controls SAC component localization is conserved also in human oocytes, we asked whether MPS1 activity was required for BUB1 and BUBR1 localization. To this aim, MII-arrested oocytes were incubated with DMSO or 500 nM reversine (n = 10/condition) for 30 minutes and then BUB1 and BUBR1 localization was analysed by immunostaining and confocal microscopy. Reversine is an aurora B kinase inhibitor^54^ and an ATP-competitive inhibitor of human MPS1^55^ with a 35-fold higher selectivity than for aurora B (IC_50_: 98 nM for aurora B vs 6 nM for MPS1)^55^. Although 500 nM reversine is the concentration used in mouse oocytes to specifically inhibit MPS1, we confirmed that at this dose aurora B activity was not affected in human oocytes^44^ by showing that phosphorylation of histone 3 on Ser10 (an aurora B phosphorylation target), was not decreased in reversine-treated oocytes (Fig. 4A). Measurement of BUB1 and BUBR1 fluorescent signal at kinetochores showed that their localization at kinetochores was significantly decreased in oocytes treated with reversine compared with controls (Fig. 4B–D), particularly for BUBR1 (three-fold lower in reversine-treated than in control oocytes) (Fig. 4D). These results indicate that like in other species, BUB1 and BUBR1 localization requires MPS1 activity also in human oocytes.

### BUB1 and BUBR1 localization at kinetochores decreases with age

**Figure 5:**
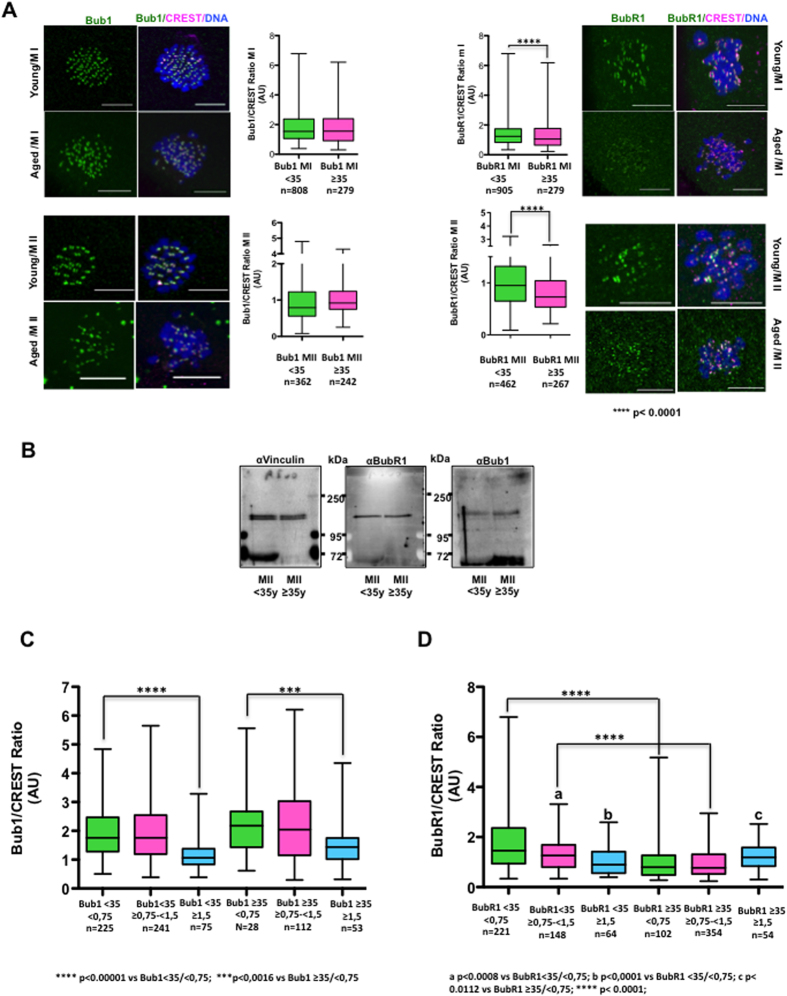
BUB1 and BUBR1 staining decreases in kinetochores from aged oocytes and in bivalents with high IKT distances. (**A**) Representative deconvolved images of MI and MII oocytes from young (<35 years) and older (≥35 years) women after immunostaining with anti-BUB1, BUBR1 and -CREST antibodies and box and whisker diagrams showing the median, minimum and maximum values of BUB1/BUBR1 to CREST immunofluorescence signal ratios. (**B**) BUBR1, BUB1 and vinculin (control) protein levels were analysed by western blotting in oocytes from young (n = 20) and older (n = 20) women. (**C**,**D**) The median, minimum and maximum levels of the BUB1-CREST and BUBR1-CREST immunofluorescence signal ratios in kinetochores classified according to their inter-kinetochore distance in oocytes from young and older women. Two-tailed unpaired Student’s *t* tests were performed to determine the statistical relevance.

We then asked whether the increased chromosome missegregation observed in oocytes from aged women correlated with decreased BUB1 and BUBR1 kinetochore localization. Quantification of BUB1 and BUBR1 signal at kinetochores in oocytes from young and older women did not highlight any significant difference in BUB1 kinetochore localization in MI and MII oocytes. Conversely, BUBR1 kinetochore signal was significantly decreased in MI and MII oocytes from older women (Fig. 5A). This was not caused by a general reduction of total BUBR1 levels as indicated by western blot analysis of oocytes from both age groups (Fig. 5B).

### BUB1 and BUBR1 kinetochore localization decreases in bivalents with high IKT distances

Our results demonstrated that IKT distances in bivalents of oocytes increased, whereas BUBR1 localization at kinetochores decreased with age. To check whether increased IKT distances of bivalents and decreased BUB1/BUBR1 kinetochore levels were correlated, the BUB1/CREST and BUBR1/CREST ratios in kinetochores from young (women <35 years) and older (women ≥35 years) oocytes classified according to their IKT distances (<0.75 μm, ≥0.75 < 1.5 μm, and ≥1.5 μm) were compared (Fig. 5C and D). The BUB1/CREST ratio was significantly higher in kinetochores with IKT distances <0.75 μm than in those with IKT ≥1.5 μm in oocytes from both young and older women (Fig. 5C). The BUBR1/CREST ratio also was significantly higher in kinetochores from young women with IKT <0.75 than in the other two IKT distance groups (Fig. 5D). These data suggest that decreased IKT cohesion reduces the ability of bivalents to retain SAC proteins at kinetochores. As expected from the results of Fig. 5A, the BUB1/CREST ratios within each IKT distance group were not significantly different between oocytes from younger and older women. Conversely, the BUBR1/CREST ratio significantly decreased in kinetochores with IKT distance of <0.75 μm and ≥0.75 < 1.5 μm in older oocytes compared with younger oocytes. This suggests that in aged oocytes, BUBR1 kinetochore localization is reduced, independently of centromere cohesion. Together these results indicate that the higher IKT distance in young oocytes results in reduced BUB1 and BUBR1 levels at kinetochores. Conversely, BUBR1, but not BUB1 levels, were generally decreased in kinetochores from aged oocytes, independently of centromere cohesion.

## Discussion

IVF techniques face to two main problems. First, 10–20% of collected oocytes cannot resume meiosis and do not progress to MII^56^,^57^. Second, oocytes collected for IVF display a high incidence of chromosomal abnormalities (60%)^58^, leading to the formation of aneuploid embryos^1^,^58^. These two main complications increase with maternal age.

In this study, first, we checked the capacity of human oocytes obtained for IVF (n = 73) to reach MII arrest. Our results demonstrate that at retrieval, oocytes from women over 35 years were mostly in GV (70.8%), whereas in women under 35 years oocytes were either in GV (44.9%) or in MI (55.1%). This significant difference in oocyte maturation between age groups was maintained after 3 hours and 24 h of incubation, suggesting that meiotic resumption is delayed in oocytes from aged women, as previously reported^59^,^60^. This could be the consequence of a differential response of older women to ovarian stimulation. It would be interesting to investigate the effect of extending the triggering duration after ovarian stimulation. It is also important to stress that the quality of the oocytes available for analysis and the hormonal treatment could have influenced the quantitative outcome. Indeed, for this study we used human oocytes that were not mature at recovering time, and that could be potentially compromised. However, this is generally the case for all studies that use human material from IVF clinics.

We then investigated the underlying mechanisms responsible of aneuploidy in human oocytes. Recent studies in mouse oocytes described loss of the cohesin complex in chromosomes and kinetochores with age^61^,^62^. This loss of cohesion could contribute to aneuploidy occurrence by promoting the disintegration of bivalents into univalents in MI^15^,^22^. If centromere cohesion is sufficiently maintained in univalents, sister kinetochores are mono-oriented and the entire univalent is pulled toward one pole, leading to MII oocytes with a normal number of chromatids. Conversely, if sister centromere cohesion is not maintained, sister chromatids become bi-oriented during MI and are pulled in the opposite directions, leading to oocytes that gain or lose one single chromatid. As it has been suggested that centromere cohesion decreases with maternal age^16^,^21^,^22^, the disintegration of bivalents into univalents during MI could explain the increase of aneuploidy in old oocytes. However, univalent formation during MI is controversial^15^,^16^,^20^,^22^. Alternatively, weak sister centromere cohesion in old oocytes could explain age-related aneuploidy, independently of univalent formation by inducing an incorrect attachment and segregation of sister kinetochores^20^. Our findings suggest that an increase of IKT distance with maternal age contributes to aneuploidy by promoting bivalent disintegration into univalents and subsequently improper sister chromatid segregation during MI. Indeed, our study shows that IKT distances are dramatically increased in aged oocytes and that this correlates with the presence of an important number of univalents in MI and of single chromatids in MII. The percentage of oocytes with at least one single chromatid is higher in aneuploid than euploid oocytes. Moreover, the number of isolated sister chromatids in MII is also significantly higher in aneuploid oocytes, particularly in aneuploid oocytes from older women. However, incorrect attachment of bivalents should lead to aneuploidy only if oocytes progress to anaphase I. Therefore, it was important to determine whether the SAC was active in these oocytes. In this study we demonstrate for the first time that the SAC proteins BUB1 and BUBR1 are localized at the kinetochores of human oocytes with the same temporal pattern as in mitotic cells. Moreover, as observed in human mitotic cells, this localization is dependent on the activity of the SAC kinase MPS1. This indicates that the signalling pathway controlling BUB1 and BUBR1 kinetochore localization is conserved and suggests that this checkpoint is active in human oocytes. In our cohort we did not detect any difference in BUBR1 protein levels when comparing oocytes according to maternal age. This result is in disagreement with a previous study showing a drop of BUBR1 protein levels in oocytes from older women^63^. We don’t know the cause of this discrepancy, however it could be explained by differences in the oocyte cohorts used in the two studies. Unfortunately, due to the limited source and the considerable number of human oocytes required to perform a western blot, there is a limited piece of data reporting BUBR1 levels in aged human oocytes. Further studies will be required to fully elucidate this issue. However, importantly, although BUBR1 protein levels were constant, we observed that BUBR1 kinetochore localization significantly decreases with age, suggesting that this checkpoint mechanism becomes less efficient with age and that this defect could be involved in maternal age-dependent aneuploidy. BUB1 and BUBR1 levels were reduced also in sister kinetochores with high IKT distances. Recent studies suggest that the stabilization of microtubule-kinetochore attachments during MI is uncoupled and independent from bivalent stretching and that it is the result of BUBR1-dependent recruitment of PP2A-B56 to kinetochores. This would promote dephosphorylation of aurora B/C substrates and the stabilization of microtubule-kinetochore attachment. Conversely, kinetochore-microtubule stabilization during MII would result from the spatial separation of aurora B/C from the attachment sites upon bivalent stretching^64^. We do not know which are the mechanisms that associate the reduction of BUB1 and BUBR1 kinetochore localization with sister centromere cohesion loss. It has been shown that increased distances of sister kinetochores during MI promotes their bipolar attachment^15^,^19^,^22^. We hypothesize that this bipolar attachment might lead to the creation of tension that spatially separates aurora B/C from the inner centromere and stabilizes microtubule-kinetochore attachments, resulting in the delocalization of SAC proteins. Due to their bipolar attachment and the subsequently created tension during MI, kinetochores of homologous chromosomes are incorrectly recognized as MII sister chromatids and the SAC is rapidly silenced in response to inter-kinetochore tension.

Together, our findings suggest that the SAC is present and active in human oocytes and that its activity decreases with the women’s age and with the IKT distance. SAC reduced activity might then contribute to the formation of univalents in which sister kinetochores form bipolar attachments that cannot be detected by the SAC, resulting in aberrant sister chromatid separation in MI and in aneuploidy.

## Methods

### Ovarian stimulation protocol and sample collection

Patients of two IVF centres (Arnaud de Villeneuve and Saint Roch Hospitals, Montpellier, France) underwent controlled ovarian stimulation, depending on the estimated ovarian response, with two gonadotropin types, r-FSH (Puregon, MSD, Courbevoie, France or GonalF, Merck Serrono, Lyon, France) or HP-hMG (Menopur, Ferring, Gentilly, France), together with a gonadotropin-releasing hormone (GnRH) agonist (Decapeptyl, IpsenPharma) or antagonist (Ganirelix Acetate, Orgalutran; MSDundergone). The agonist was administered daily. Ovulation was triggered by injection of 250 μg human chorionic gonadotropin (hCG) (Ovitrelle, Merck Srno, Lyon, France) when at least three follicles reached the diameter of 17 mm or more on ultrasound examination. Oocytes were retrieved by transvaginal ultrasound-guided aspiration 36 h after hCG injection and used for IVF by intracytoplasmic sperm injection (ICSI). In total, 226 immature oocytes from 142 patients who underwent ICSI for male infertility (40.07%), mechanical infertility (7.96%), pre-implantation genetic diagnosis (42%), idiopathic infertility (7.96%) or endometriosis (1.38%) between February 2014 and October 2016 were analysed in this study. The women’s age was 32.6 ± 4.2 years (mean ± SD; range: 22 to 42).

The study followed the relevant regulatory standards and was approved by the French Biomedicine Agency. All participants were informed about the study and signed a written informed consent form for the use of discarded oocytes before enrolment.

### Oocyte preparation

Within 1–3 h from retrieval, cumulus cells were removed. Immature oocytes were recovered for use in this study. Oocytes were transferred in microdrops of G1-PLUS medium (Vitrolife Ltd, United Kingdom) and incubated at 37 °C in 5% CO_2_ humidified atmosphere for another 2 hours.

Oocyte morphology was first assessed under a phase-contrast microscopy. Depending on the development stage at this time, oocytes were: (i) immediately fixed and stored at 4 °C for immunofluorescence analysis; (ii) incubated for 16 to 30 hours to reach metaphase I or metaphase II (polar body extrusion) and then fixed and stored at 4 °C for staining or washed in PBS, snap-frozen and stored at −80 °C for immunoblotting; (iii) incubated with reversine, fixed and stored at 4 °C for staining.

### Oocyte fixation and immunostaining

Oocyte fixation and storage were adapted from Coticchio *et al*.^65^. Briefly, oocytes were fixed in PHEM buffer (25 mM HEPES, 60 mM PIPES, 2 mM MgCl2, 10 mM EGTA) containing 2% formaldehyde, 0.1% Triton-X-100 and 10 U/ml aprotinin at 37 °C for 30 min and stored in blocking solution (2% normal goat serum, 2% normal donkey serum, 1% BSA, 0.1 M glycine, 0.2% sodium azide, and 0.1% Triton X-100 in PBS) at 4 °C until further processing. For improving kinetochore visualization, oocytes were permeabilized in a drop of PHEM buffer with 0.25% Triton-X-100 before fixation. After storage, fixed oocytes were incubated at 4 °C with primary antibodies diluted in blocking solution overnight. Oocytes were washed three times in blocking solution and then incubated at 37 °C with secondary antibodies and Hoechst 33342 (Molecular probes) for 2 hours. Following several washes, oocytes were mounted in Citifluor AF1 (Citifluor Ltd, England) with minimal compression using a hole reinforcement label.

Primary antibodies included: home-made affinity-purified rabbit anti-human BUB1 (1:250), mouse anti-human BUBR1 (1:250; BD Biosciences, United States), a mixture of mouse anti-alpha tubulin (1:400) and mouse anti-beta tubulin (1:800) (Sigma) or rabbit anti-alpha tubulin (1:500) (gift from Jose Manuel Andreu, CIB, Spain), anti-phosphorylated H3 Ser10 rabbit polyclonal antibody (Millipore) and human anti-centromere CREST autoantibody (1:500) (FZ90C-CS1058, Europa Bioproducts Ltd, United Kingdom). Secondary antibodies were Alexa-Fluor-633-conjugated anti-rabbit (1:500), Alexa-Fluor-546-conjugated anti-mouse (1:800), Alexa-Fluor-633-conjugated anti-mouse (1:500), Alexa-Fluor-546-conjugated anti-rabbit (1:800) and Alexa-Fluor-488-conjugated anti-human (1:1000) (all Molecular Probes, ThermoFisher Scientific, United States). All the other reagents were from Sigma (Sigma-Aldrich, Belgium).

### Generation and purification of the home-made anti-human BUB1 antibody

Anti-human BUB1 (hBUB1) antibodies were generated by immunizing rabbits with a purified glutathione *S*-transferase (GST) fusion protein that corresponded to amino acids 1–283 of hBUB1. Serum was affinity-purified on immobilized GST-hBUB1 fusion protein.

### Cell culture and SAC activation

Human HeLa cells were grown in DMEM medium with 10% FBS and penicillin/streptomycin (complete medium). For SAC activation, HeLa cells were synchronized in complete medium containing 2.5 mM thymidine for 24 hours, then released in complete medium supplemented with 25 μM 2′-deoxycytidine for 3 hours and finally incubated in complete medium with DMSO (control) or 90 ng/ml nocodazole (mitotic block) for 12 hours. Cells were then lysed, immunoprecipitated and immunoblotted as indicated.

### Reversine treatment

Twenty MII-arrested (polar body presence for at least 6 hours, or 27 hours after GV breackdown) oocytes (from 10 patients) were washed three times in G1-PLUS medium containing DMSO (control) or 500 nM reversine in DMSO. Oocytes were then incubated in 25 μl-drops of DMSO or 500 nM reversine in mineral oil at 37 °C in a CO_2_ incubator for 30 min. Oocytes were then fixed for immunostaining, as described.

### Image acquisition

Images of fixed oocytes were acquired using a Leica SP8 confocal microscope (Leica Microsystems, Germany) equipped with a Leica 63x HCX PL APO 1.4 oil immersion objective and HeNe (633 nm excitation), KrArg (488 and 561 nm excitation) and diode 405 (405 nm excitation) lasers for collection of complete four channel Z-stacks through the entire spindle of each oocyte. Optical sections were collected at 0.3 μm intervals. Images of oocytes for fluorescence intensity measurements were all acquired using the same technical conditions.

### Image analysis

Images of entire meiotic spindles or DNA surfaces (for intensity measurements) were deconvolved using Huygens Professional (Scientific Volume Imaging) and then further analysed with Image J (NIH, USA) and Imaris (Bitplane, Switzerland). To determine the distance between two sister chromatid kinetochores, the automated spot detection function in Imaris was used to detect each kinetochore (CREST staining), followed by visual observation and manual correction of software errors in MI oocytes. The spot to spot closest distance function (interface with Matlab) was then used to determine the minimum distance between spots. Pairings were then controlled visually by locating CREST spots on DNA in 3D images. Measurement data were included in the study only if they belonged clearly to a sister kinetochore pair within a bivalent (70 to 100% of kinetochore pairs). In MII-arrested oocytes, sister chromatids were detected manually and IKT distances were calculated using the Pythagorean Theorem. To determine BUB1/BUBR1 and CREST signal intensity at kinetochores, the automated spot detection function in Imaris was used in the BUB1/BUBR1/CREST detection channels. BUB1/BUBR1 and CREST spots were colocalized and visual observation allowed correcting software errors. The BUB1/BUBR1 mean signal intensities in the BUB1/BUBR1 and CREST spots were exported to Excel files for further analysis.

### Western blot analysis

Snap-frozen oocytes were lysed and pooled by 20 according to their maturation stage or to women’s age directly in Laemmli sample buffer containing DTT as reducing agent. Samples were boiled and proteins were resolved on 8% SDS-PAGE gels. Membranes were blocked at room temperature (RT) in 5% skimmed milk/TBS-Tween 20 for 1 hour, incubated with anti-hBUBR1 (1:500) and anti-BUB1 (1:1000) at 4 °C overnight, washed three times, and then incubated with IRDye 680/800-conjugated antibodies (ThermoScientific) at RT for 1 hour. Loading was checked with an anti-beta-actin or anti-vinculin antibody (Sigma-Aldrich, Belgium). Detection was performed with the Odyssey Infrared Imaging System (LI-COR Biosciences).

### Statistical analysis

The means (±SD) were calculated using Microsoft Excel. Unless otherwise stated, unpaired two-tailed t-tests were used to assess differences using the Prism5 GraphPad or Microsoft Excel software programs. Linear regression analysis was performed with Prism5 GraphPad.

## Additional Information

**How to cite this article**: Lagirand-Cantaloube, J. *et al*. Loss of Centromere Cohesion in Aneuploid Human Oocytes Correlates with Decreased Kinetochore Localization of the Sac Proteins Bub1 and Bubr1. *Sci. Rep.* **7**, 44001; doi: 10.1038/srep44001 (2017).

**Publisher's note:** Springer Nature remains neutral with regard to jurisdictional claims in published maps and institutional affiliations.

## Supplementary Material

Supplementary Information

Supplementary Video 1

Supplementary Video 2

Supplementary Video 3

Supplementary Video 4
