# Supplementary material for: Loss of Centromere Cohesion in Aneuploid Human Oocytes Correlates with Decreased Kinetochore Localization of the Sac Proteins Bub1 and Bubr1
Source: Sci Rep. 2017 Mar 13;7:44001. doi: 10.1038/srep44001 (PMC5347135; doi:10.1038/srep44001)
Supplement: Supplementary Information [file srep44001-s1.pdf]

Supplementary Information

LOSS OF CENTROMERE COHESION IN ANEUPLOID HUMAN OOCYTES CORRELATES WITH DECREASED KINETOCHORE LOCALIZATION OF THE SAC PROTEINS BUB1 AND BUBR1

Julie Lagirand-Cantaloube[1,2], Cendrine Ciabrini[2], Sophie Charrasse[1], Alice Ferrieres[3], Anna Castro[1], Tal Anahory[1,2,4*] and Thierry Lorca[1*].

Supplementary Fig. 1 Lagirand-Cantaloube et al.

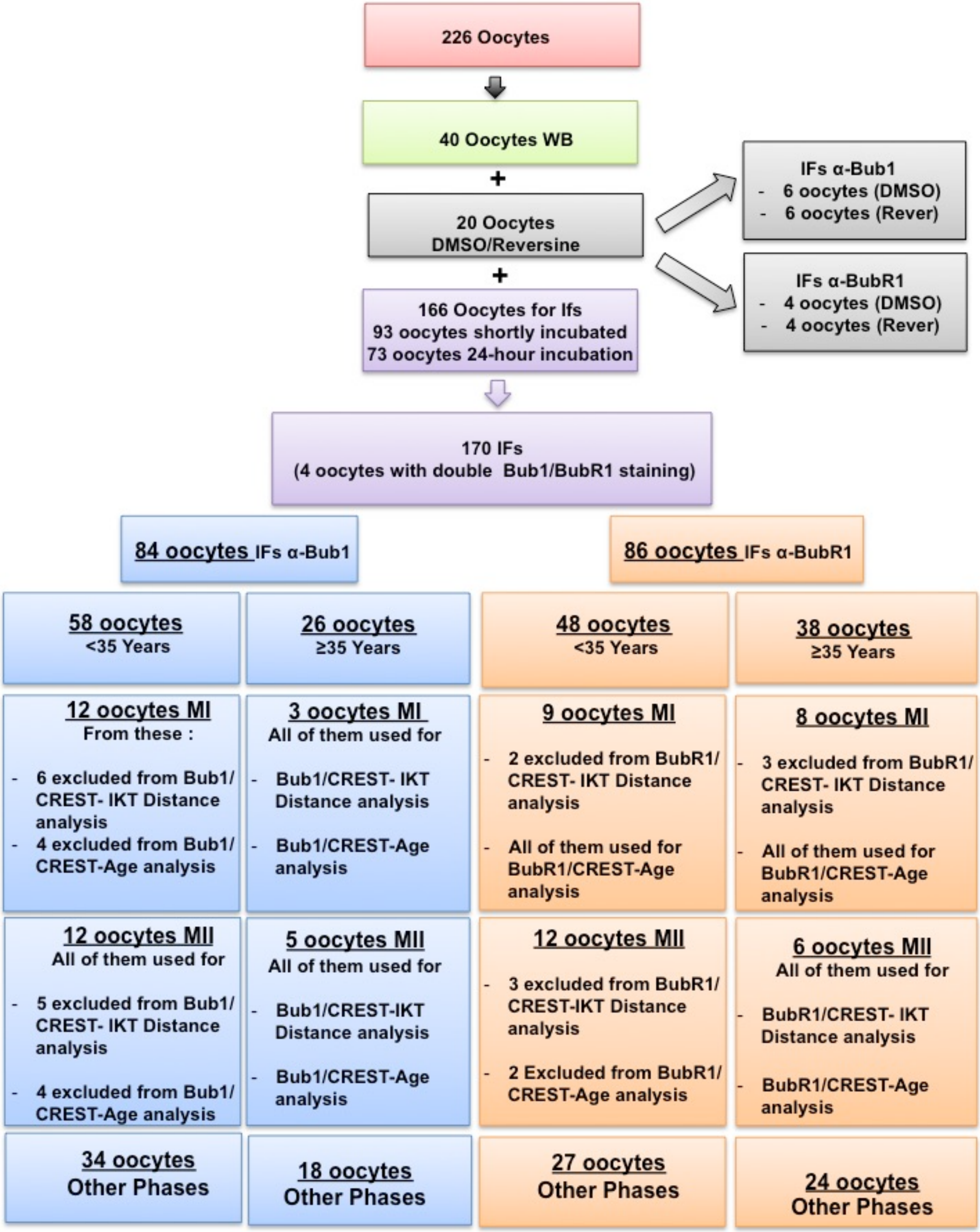

Diagram detailing the analysis performed in each oocyte used in this study

Supplementary Fig. 2 Lagirand-Cantaloube et al.

BUB1 IMMUNOFLUORESCENCE

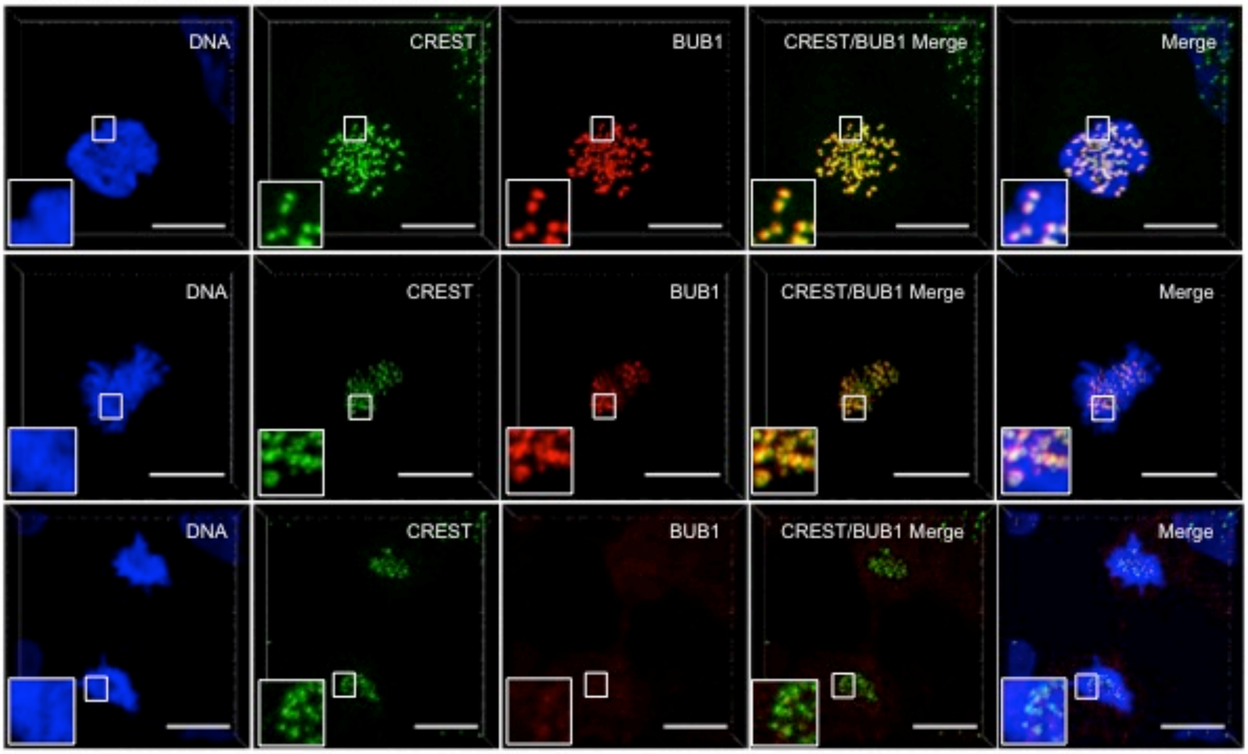

BUBR1 IMMUNOFLUORESCENCE

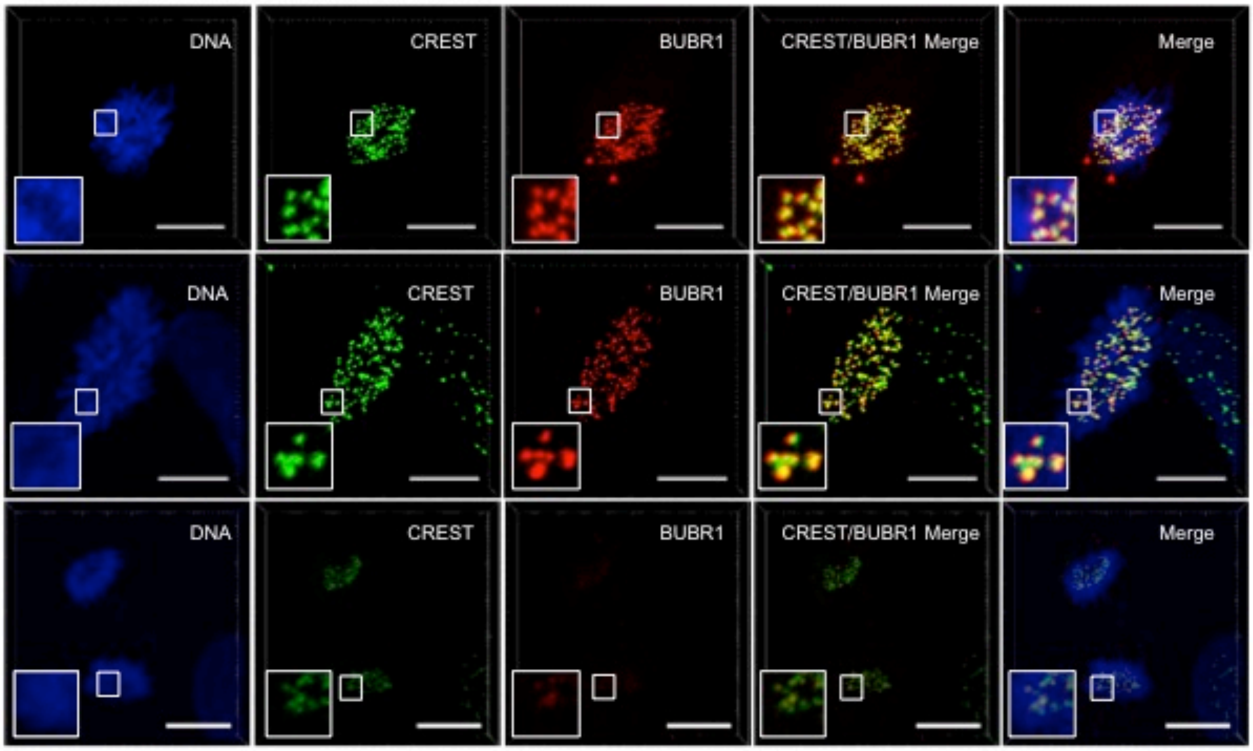

U2OS cells were used for confocal immunofluorescence using anti-Bub1, -BubR1 and anti-CREST antibodies and Hoechst for DNA staining. Shown are zooms of the boxed area using ImageJ. Bars 10 μm. Z-stacks 200 nm were recorded on a confocal Leica SP5-SMD. Images were processed using Imaris software (Bitplane).

Supplementary Fig. 3 Lagirand-Cantaloube et al.

A

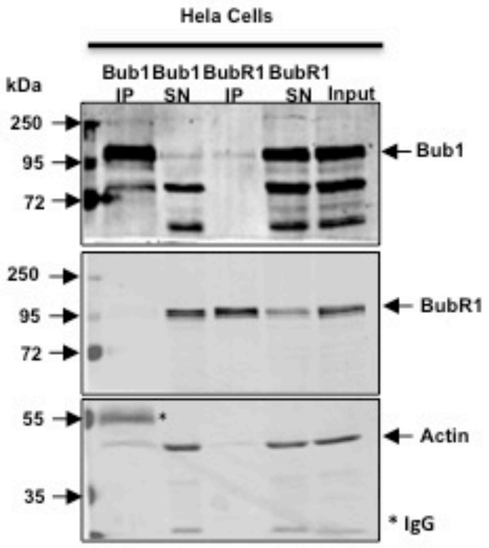

Mitotic arrested HeLa cell extracts were used to perform immunoprecipitation using anti-Bub1 and anti-BubR1 antibodies. Input, immunoprecipitates (IP) and supernatants (SN) were loaded. Both antibodies specifically immunoprecipitate and recognise Bub1 and BubR1 proteins respectively. Actin was used as a loading control.

B

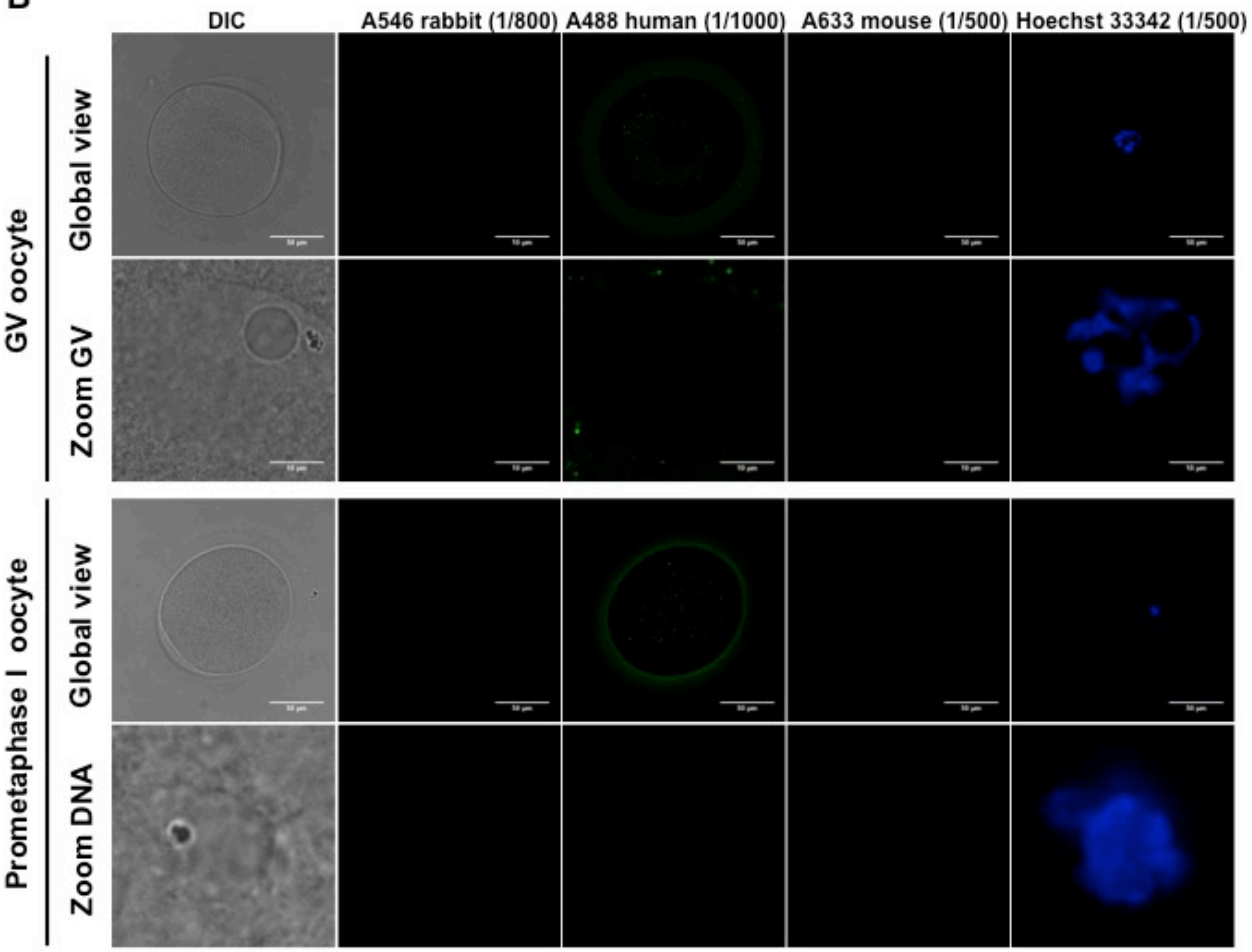

Background signal checked by immunofluorescence by incubating oocytes with the secondary antibodies.

Supplementary Figure 4 Lagirand-Cantaloube et al.

A

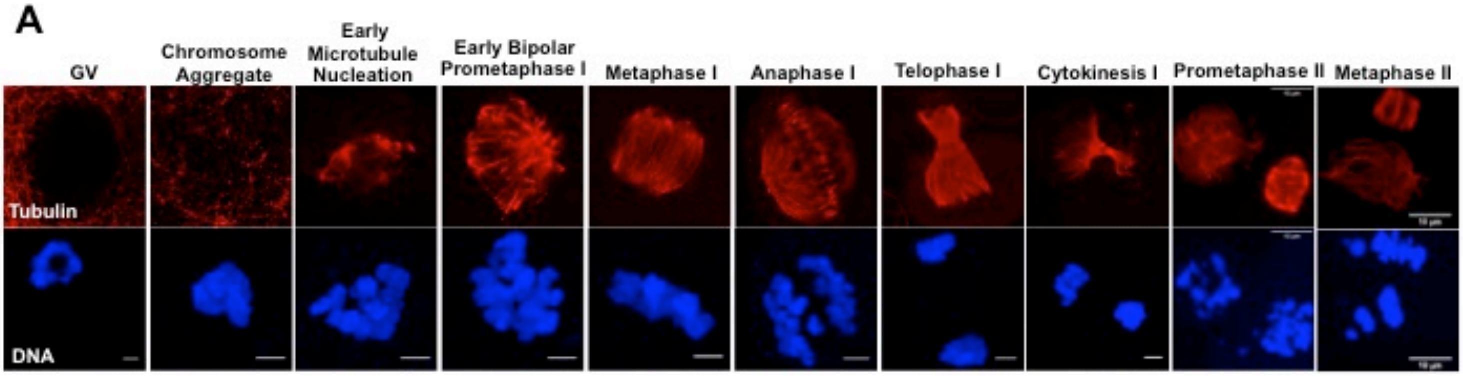

Representative images of oocytes at the different meiotic stages. Bar 10 μm

B

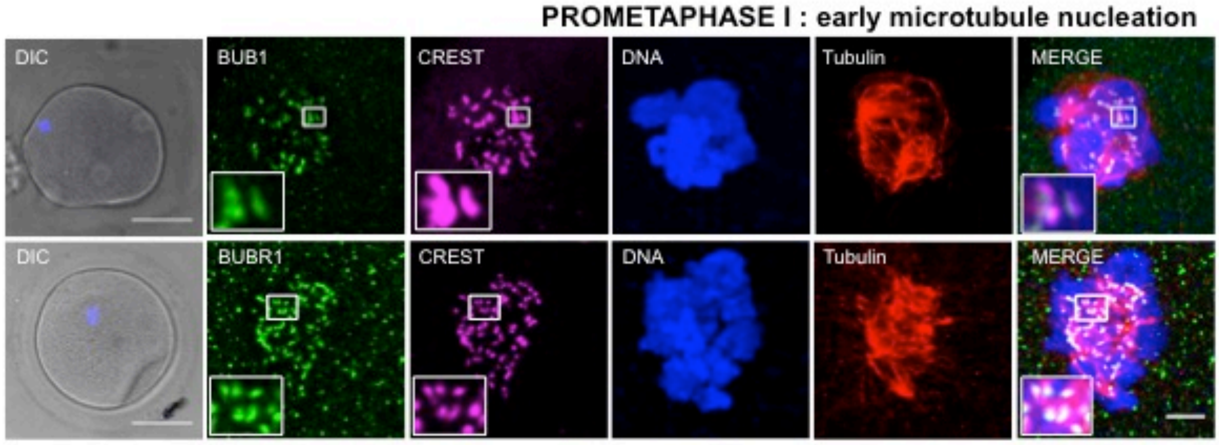

C

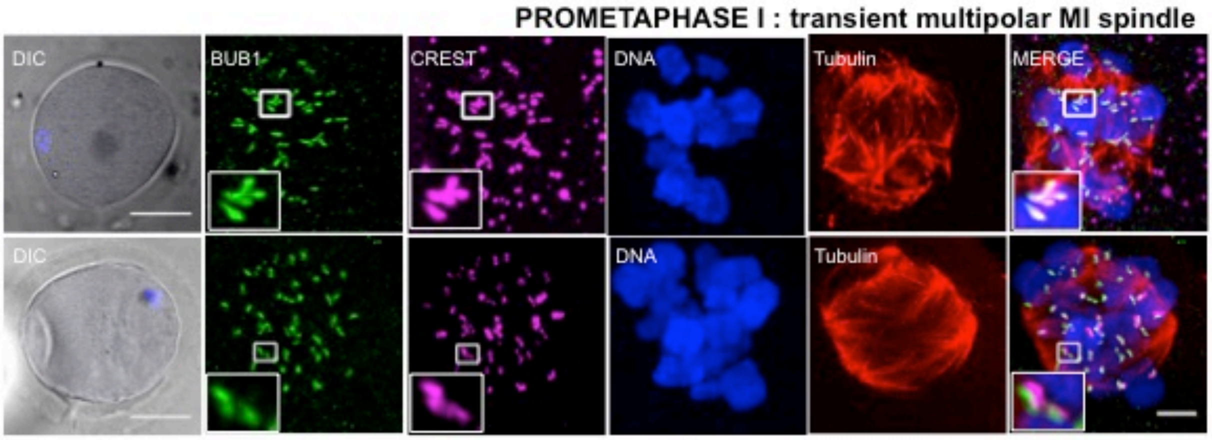

Representative deconvolved images of oocytes immunostained with anti-Bub1 and anti-BubR1 antibodies in prometaphase I:early microtubule nucleation and **(B)**, in prometaphase I: transient multipolar MI spindle **(C)**. Shown are zooms of the boxed area using ImageJ. Bars 5 μm.

Supplementary Figure 5 Lagirand-Cantaloube et al.

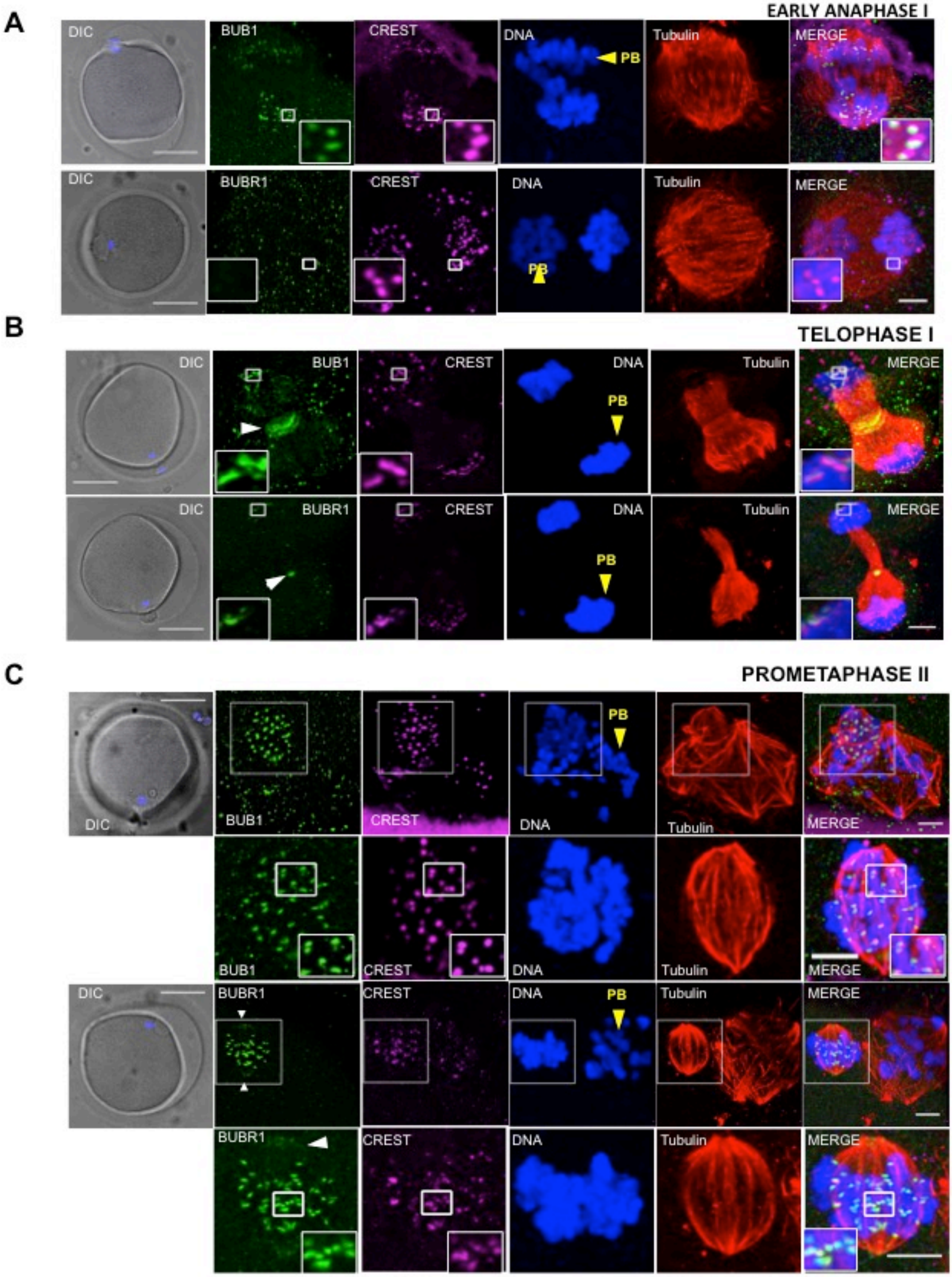

Representative deconvolved images of oocytes immunostained with anti-Bub1 and anti-BubR1 antibodies in early anaphase I **(A)**, in telophase I **(B)** and prometaphase II **(C)**. For (A) and (B) the boxed areas are zoomed using ImageJ. For (C), second and fourth rows correspond to zooms of the boxed areas of the first and third row. Bars 5 μm.

Video Legends

Legend to Video 1: MI oocyte with normal bivalent distribution. Spots corresponding to kinetochores were automatically detected with Imaris software in deconvolved images of oocytes immunostained with Hoetchst (blue) and anti-CREST antibodies (magenta). Video obtained from Imaris allowing the visualization of the metaphase plate in 3D. Scale bar, 5 μm.

Legend to Video 2: MI oocyte with univalent. As for Video 1, except that univalent is shown by an arrowhead. Scale bar, 5 μm.

Legend to Video 3: MI oocyte displaying a single chromatid. Video showing 3D-rotation of a MI oocyte with a single chromatid (arrowhead). Scale bar, 5 μm.

Legend to Video 4: MII oocyte showing single chromatids. 3D rotation video of a MII oocyte displaying four isolated sister chromatids. Three of these sister chromatids are pointed out by white arrowheads. Scale bar, 5 μm.
